# Hydroxypropyl Methylcellulose (HPMC) Edible Films Incorporated with Melatonin: Physicochemical Properties and Quality Preservation of Coated Green Asparagus (*Asparagus officinalis* L.) During Cold Storage

**DOI:** 10.3390/foods15213856

**Published:** 2026-10-28

**Authors:** Pontree Itkor, Athip Boonsiriwit

**Affiliations:** 1 Department of Food Science and Technology, Thammasat University, Pathum Thani 12120, Thailand; pontree.itkor@gmail.com; 2 Center of Excellence in Food Science and Innovation, Thammasat University, Pathum Thani 12120, Thailand

**Keywords:** coating treatment, edible film, green asparagus, HPMC, melatonin, enzymatic activity

## Abstract

Green asparagus (*Asparagus officinalis* L.) has a short shelf life due to its high respiration rate, leading to rapid senescence, weight loss, and toughening after harvest. While melatonin (ML) is known to delay postharvest senescence, its application is limited by environmental instability. This study developed hydroxypropyl methylcellulose (HPMC)-based edible films incorporating ML (0, 25, 50, and 100 µM) and evaluated their physicochemical properties. The corresponding formulations were applied as edible coatings to the basal region of asparagus spears to evaluate their preservation performance during 25 days at 4 °C and 85% relative humidity (RH). The incorporation of ML reduced the water vapor transmission rate (WVTR) without compromising the mechanical properties of the films. Compared with uncoated green asparagus, HPMC/ML coatings suppressed the activities of lignification-related enzymes, phenylalanine ammonia-lyase (PAL) and peroxidase (POD), thereby attenuating postharvest toughening. Furthermore, the HPMC/ML treatment enhanced the activities of antioxidant-related enzymes, catalase (CAT) and superoxide dismutase (SOD), which were associated with reduced hydrogen peroxide (H_2_O_2_) accumulation. The HPMC/ML coating also reduced weight loss, retained chlorophyll content, and attenuated the change in firmness of the storage green asparagus. Considering the overall properties, HPMC/ML50 was identified as the recommended formulation for delaying postharvest senescence and maintaining green asparagus quality during cold storage.

## 1. Introduction

Asparagus (*Asparagus officinalis* L.), a non-climacteric vegetable belonging to the Asparagaceae family, is globally recognized as a premium commodity due to its unique flavor, texture, and abundant content of bioactive substances, including vitamins, amino acids, and polyphenols [1,2]. Green asparagus, primarily consumed fresh, is known for its crisp texture and unique flavor; however, it has a limited shelf life after harvest due to its high metabolic rate and rapid respiratory activity. This results in significant quality deterioration during storage, with undesirable physiological and compositional changes, including weight loss, yellowing, toughening, and the rapid degradation of nutrients and bioactive compounds [3,4].

Asparagus texture is one of the critical attributes for consumer acceptance and is directly related to freshness. Postharvest deterioration of asparagus is characterized by an increase in firmness, followed by wrinkling and severe toughening due to lignification [5]. This complex process involves the synthesis, deposition, and polymerization of lignin monomers, commencing with the phenylpropanoid pathway, regulated by key enzymes such as phenylalanine ammonia-lyase (PAL) and peroxidase (POD). After synthesis, lignin is transported to the secondary cell wall and forms complex polymers, leading to hardening [4,5]. In addition to textural deterioration, the senescence of asparagus is visually apparent through color change resulting from chlorophyll degradation [6]. These changes, together with moisture loss and oxidative deterioration, progressively reduce the visual quality and marketability of asparagus during postharvest storage. Consequently, various postharvest preservation techniques, such as chemical treatments, edible coatings, and innovative packaging technologies have been adopted to mitigate these adverse changes and extend the shelf life of asparagus [7,8,9]. Among these approaches, the application of bioactive compounds capable of regulating senescence-related processes has attracted increasing attention.

Melatonin (ML; N-acetyl-5-methoxytryptamine) has emerged as a promising bioactive compound for mitigating postharvest quality deterioration. This low-molecular-weight tryptophan derivative is a natural indoleamine found in both animals and plants. ML plays a significant role in plant development by promoting growth, delaying leaf senescence, and facilitating fruit maturity. Exogenous ML treatment has been widely investigated for delaying senescence, alleviating chilling injury, and suppressing chlorophyll degradation, thereby preserving the quality of various fresh produce [10,11,12,13]. Furthermore, ML functions as a promising antioxidant, which can directly scavenge reactive oxygen species (ROS) and enhance the intrinsic antioxidant system [6,14]. Previous research has shown that ML application effectively reduces lignification in green asparagus by regulating PAL and POD activity, thereby extending postharvest shelf life [15]. Despite these beneficial effects, ML stability can be influenced by environmental factors, such as light, temperature, and storage time, which significantly limit its practical efficacy and commercial application, necessitating the development of a stable and protective delivery system [15,16].

In this context, edible coatings based on biopolymers offer a promising approach for incorporating bioactive compounds into food preservation systems. Hydroxypropyl methylcellulose (HPMC) is particularly advantageous due to its excellent film-forming properties, high optical transparency, and proven efficacy as an active compound carrier without altering the sensory profile of the food. Furthermore, HPMC provides a semi-permeable barrier to regulate moisture and gas exchange, offering potential benefits for preserving highly respiring produce like asparagus [17,18]. The incorporation of ML into the HPMC matrix may therefore combine the physical barrier function of the coating with the physiological effects of ML in delaying postharvest senescence. However, because the incorporation of bioactive compounds may also influence the physicochemical characteristics and barrier performance of the polymer matrix, evaluating these properties is essential alongside their effectiveness in preserving fresh produce.

Therefore, this study aimed to develop HPMC-based edible films incorporating ML (HPMC/ML) and characterize their physicochemical properties, including thickness, mechanical strength, transparency, oxygen transmission rate (OTR), and water vapor transmission rate (WVTR). Subsequently, the HPMC/ML formulations were applied as edible coatings to green asparagus to investigate their effectiveness in preserving postharvest quality during cold storage. Changes in key quality attributes, including appearance, color, weight loss, texture, chlorophyll content, and lignin accumulation, were systematically monitored. In addition, hydrogen peroxide (H_2_O_2_) content and the activities of lignification-related enzymes (PAL and POD) and antioxidant-related enzymes (catalase (CAT) and superoxide dismutase (SOD)) were evaluated to investigate the physiological and biochemical responses associated with the coating treatments. The findings were expected to provide insights into the potential of HPMC/ML coatings for delaying postharvest senescence and maintaining the quality of green asparagus during refrigerated storage.

## 2. Materials and Methods

### 2.1. Materials

Hydroxypropyl methylcellulose (HPMC) was purchased from Alfa Aesar (Ward Hill, MA, MA, USA). Melatonin (ML, ≥98%) was obtained from Tokyo Chemical Industry Co., Ltd. (Tokyo, Japan). All the other chemical reagents used were of analytical grade.

### 2.2. Preparation and Characterization of Hydroxypropyl Methylcellulose Edible Films Incorporated with Melatonin (HPMC/ML)

#### 2.2.1. Preparation of HPMC/ML Films

HPMC/ML films were prepared using the solvent casting technique. A 4% (*w*/*w*) HPMC solution was prepared by dissolving HPMC in distilled water at 80 °C, followed by homogenization. The solution was cooled to 40 °C before the incorporation of ML. ML was separately dissolved in the minimum amount of 70% (*v*/*v*) ethanol, only to achieve complete dissolution at each target concentration. The resulting ML solutions were subsequently mixed with the HPMC solution to obtain final ML concentrations in the liquid film-forming solutions of 25, 50, and 100 µM (designated as HPMC/ML25, HPMC/ML50, and HPMC/ML100, respectively), whereas the HPMC formulation without ML was used as the control. No additional plasticizer was included in any formulation. These formulations were also used for the subsequent coating treatments. The mixture was degassed using a sonicator at 40 °C for 30 min to remove the entrapped air bubbles. A 20 mL aliquot of each solution was then cast into polystyrene square dishes (12 cm × 12 cm) and dried at 25 °C for 24 h. The resulting dried films were peeled, wrapped in aluminum foil, and stored in a desiccator at room temperature (25 °C) until further characterization.

#### 2.2.2. Characterization of HPMC/ML Edible Film

##### Mechanical Properties

Film thickness was measured using a micrometer (Mitutoyo, Kawasaki, Japan). The tensile strength (TS) and elongation at break (EAB) of the films were determined using a universal testing machine according to GB 13022-1991. Before testing, the films were cut into rectangular strips (6.0 cm × 2.0 cm) and conditioned at 25 °C and 50% relative humidity (RH) for 24 h. The universal testing machine was equipped with a 10 kgf load cell, and the initial grip separation was set to 4.0 cm. The test speed was maintained at 0.06 mm/s, and five measurements were performed for each formulation.

##### Barrier Properties

The oxygen transmission rate (OTR) and water vapor transmission rate (WVTR) of the films were evaluated following the previously reported method [19]. Briefly, the OTR of the films was evaluated using an 8001 Oxygen Permeation Analyzer (Illinois Instruments, Inc., Johnsburg, IL, USA) at 23 °C and 0% RH. The WVTR was determined using a 7000 Water Vapor Permeation Analyzer (Illinois Instruments, Inc., Johnsburg, IL, USA) at 38 °C and 90% RH. These controlled testing conditions enabled comparative assessment among the HPMC/ML film formulations.

##### Optical Properties

Film transparency was determined by measuring the absorbance at 600 nm (A600) using a UV-vis spectrophotometer (V-650, JASCO Corporation, Hachioji, Japan) and calculated using the equation [19]:

(1) Transparency (Abs/mm)=A600/e,

where *e* is the film thickness (mm).

### 2.3. Application of HPMC/ML as Edible Coating on Green Asparagus

#### 2.3.1. Preparation of Green Asparagus

Fresh green asparagus spears (*Asparagus officinalis* L.) were obtained from a local farm in Thailand and transported to the laboratory within 2 h after collection. Upon arrival, the spears were precooled at 4 °C for 4 h and selected for uniformity based on diameter (1.2–1.5 cm), length (approximately 20 cm), straightness, and absence of visible damage. The selected asparagus spears were subsequently randomly divided into batches of 300 g each.

#### 2.3.2. Application of HPMC/ML Edible Coating

The coating procedure was carried out at room temperature by dipping the basal part (10 cm of the spears) of the asparagus into the respective coating solutions (HPMC, HPMC/ML25, HPMC/ML50, and HPMC/ML100). Following dipping, the spears were immediately withdrawn from the coating solutions without a separate draining step. Subsequently, the asparagus spears were allowed to air-dry in a laminar-flow chamber at 25 °C and 50% RH, without additional forced airflow, for 40 min before storage. Control samples were similarly dipped in distilled water to account for the effects of dipping, handling, and drying procedures. Each batch of asparagus was placed in a perforated plastic basket, with the spears standing upright. All the samples were stored at 4 °C under 85% RH, and sampling was performed to analyze the quality parameters on days 0, 5, 10, 15, 20, and 25 of storage.

#### 2.3.3. Determination of Green Asparagus Quality Parameters During Cold Storage

##### Weight Loss

Weight loss was calculated as the percentage difference between the initial weight (*W*_0_) and the weight at time t (*W_t_*) using the equation:

(2) $$Weight\;loss\;(\%)={(W}_{0}-W_{t})/W_{0}\times 100,$$

##### Color

The external color of the asparagus was measured using a Colorimeter (Konica Minolta, Inc., Sakai, Japan). Measurements were performed at the middle region of the asparagus spears to provide a consistent measurement location and minimize variability arising from morphological and pigmentation differences along the spear. Ten asparagus spears were analyzed per treatment. The total color difference (Δ*E*) was calculated using the equation:

(3) $$∆E=\;\sqrt{{\left(L^{*}-L_{0}^{*}\right)}^{2}+{\left(a^{*}-a_{0}^{*}\right)}^{2}+{\left(b^{*}-b_{0}^{*}\right)}^{2}},$$

where *L*_0_*, *a*_0_*, and *b*_0_* are the color parameters related to the initial color of asparagus, and L*, a*, and b* are the color values of asparagus on different days of storage.

##### Texture

The texture of green asparagus was evaluated by a cutting test using a TA1 Texture Analyzer (Lloyd Instruments, AMETEK, Inc., Largo, FL, USA) equipped with a Warner–Bratzler Shear Blade Set (V-shaped blade; FG/SBS, part no. 01/2767), following the method described by Boonsiriwit, Lee, Kim, Itkor and Lee [15]. The measurement was performed at the basal region, 3 cm from the base of each asparagus spear, with a constant crosshead speed of 5 mm/s and a cutting depth of 5 mm. The firmness was determined from the maximum cutting force (N), and the results were expressed as mean values of ten replicates.

##### Lignin Content

The lignin content was determined following the method of Fukushima and Hatfield [20]. Fresh asparagus tissue (1 g) was homogenized in 5 mL of 95% (*v*/*v*) ethanol and centrifuged at 10,000× *g* for 10 min at 4 °C. The resulting residue was washed three times with 3 mL of ethanol:hexane solution (1:2, *v*/*v*) to remove extractable phenolic compounds and lipids. Subsequently, the residue was treated with 1 mL of acetyl bromide–acetic acid solution (1:3, *v*/*v*) and incubated in a water bath at 50 °C for 2 h. After cooling, the mixture was centrifuged at 3000× *g* for 15 min. An aliquot (0.5 mL) of the digested solution was mixed with 6.5 mL of acetic acid and 2 mL of 0.3 M NaOH, followed by the addition of 1 mL of 0.5 M hydroxylamine hydrochloride. After thorough mixing, the absorption was measured at 280 nm using a UV-vis spectrophotometer (V-650, JASCO Corporation, Hachioji, Japan). Quantification of the lignin content was carried out using a standard coumaric acid curve, with the results expressed as g lignin/kg fresh weight.

##### Chlorophyll Content

Total chlorophyll content was determined following the method described by Li and Zhang [21]. Briefly, 1 g of chopped asparagus tissue was homogenized with 20 mL of 80% acetone at a moderate speed for 30 s. Following homogenization, the mixture was quantitatively transferred into a 50 mL volumetric flask using 80% acetone and made up to volume with the same solvent. The homogenate was filtered and centrifuged at 10,000× *g* for 15 min at 4 °C. The absorbance of supernatant was recorded at 647 nm (A647) and 664.5 nm (A664.5) using a UV–vis spectrophotometer (V-650, JASCO Corporation, Hachioji, Japan). Total chlorophyll content was calculated using Equation (4) and expressed as mg/100 g fresh weight (FW).

(4) Chlorophyll content (mg/100 g fresh weight)=5×(17.95×A647+8.08×A664.5),

##### Hydrogen Peroxide (H_2_O_2_) Content

Hydrogen peroxide (H_2_O_2_) content was determined according to the method described by Jana and Choudhuri [22]. Briefly, asparagus tissue (5 g) was homogenized in 50 mL of 50 mM phosphate buffer (pH 6.5), followed by centrifugation at 10,000× *g* for 30 min at 4 °C. Subsequently, 3 mL of the supernatant was mixed with 1 mL of 0.1% titanium sulfate prepared in 20% (*v*/*v*) H_2_SO_4_. The absorbance of the resulting complex was measured at 480 nm using a UV–vis spectrophotometer. H_2_O_2_ concentration was determined using a standard calibration curve and expressed as mg/100 g fresh weight.

##### Enzyme Activity Assays

The enzyme activities, including phenylalanine ammonia-lyase (PAL), peroxidase (POD), catalase (CAT), and superoxide dismutase (SOD), were determined using spectrophotometric methods on crude extracts prepared from fresh asparagus tissue. Protein concentration in the crude enzyme extracts was determined using the Bradford method with bovine serum albumin (BSA) as the standard [23]. Specific enzyme activities were expressed as units per milligram of protein (U/mg protein).

PAL activity was measured following Wang et al. [24]. Briefly, 2 g of fresh tissue samples were homogenized with 6 mL of 50 mM Tris-HCl buffer (pH 8.8) containing 15 mM β-mercaptoethanol, 5 mM EDTA, 5 mM ascorbic acid, and 0.15% (*w*/*v*) polyvinylpyrrolidone (PVP). The resulting homogenate was centrifuged at 12,000× *g* for 30 min at 4 °C. The supernatant was collected as the crude enzyme extract. 3 mL reaction mixture, containing 16 mM l-phenylalanine, 50 mM Tris-HCl buffer (pH 8.9), 3.6 mM NaCl, and 0.5 mL of the crude enzyme extract, was incubated at 37 °C for 1 h. The reaction was stopped by adding 500 µL of 6 M HCl. PAL activity was determined by measuring the increase in absorbance at 290 nm associated with trans-cinnamic acid formation. One unit (U) of PAL activity was defined as the amount of enzyme producing an increase in absorbance of 0.01 per hour at 290 nm.

POD enzyme activity was determined following the method described by Zhang et al. [25]. A 2 g aliquot of the tissue sample was homogenized with 10 mL of 50 mM sodium phosphate buffer (pH 7.0) containing 0.2 g of PVP. Following centrifugation at 12,000× *g* for 30 min at 4 °C, the supernatant was collected as the crude enzyme extract. 3 mL reaction mixture comprised 25 µL of crude enzyme extract, 2.73 mL of 50 mM phosphate buffer (pH 7.0), 0.10 mL of 1% H_2_O_2_ solution, and 0.15 mL of 4% guaiacol solution. POD activity was determined by monitoring the increase in absorbance at 470 nm due to guaiacol oxidation over 2 min. One U of POD activity was defined as the amount of enzyme producing an increase in absorbance of 0.001 per minute at 470 nm.

CAT activity was measured according to the method described by Li et al. [26], with slight modifications. 2 g of asparagus tissue was homogenized with 10 mL of 0.2 M phosphate buffer (pH 7.8) containing 1% (*w*/*v*) PVP. The homogenate was centrifuged at 12,000× *g* for 30 min at 4 °C, and the supernatant was collected as the crude enzyme extract. The reaction mixture contained 0.2 mL of crude enzyme extract, 0.3 mL of 0.1 M H_2_O_2_, 1.5 mL of phosphate buffer (pH 7.0), and 1.0 mL of distilled water. CAT activity was determined by monitoring the decrease in absorbance at 240 nm associated with H_2_O_2_ decomposition. One U of CAT activity was defined as the amount of enzyme producing a decrease in absorbance of 0.1 per minute at 240 nm.

SOD activity was determined following the methods described by Li, Zhang and Wang [26] and Tang et al. [27]. 2 g tissue sample was homogenized with 10 mL of 50 mM phosphate buffer containing 0.1 mM EDTA, 0.3% (*w*/*v*) Triton X-100, and 4% (*w*/*v*) polyvinylpyrrolidone (PVP). The homogenate was centrifuged at 12,000× *g* for 30 min at 4 °C, and the supernatant was collected as the crude enzyme extract. SOD activity was subsequently determined spectrophotometrically at 525 nm based on the inhibition of nitroblue tetrazolium (NBT) reduction. One U of SOD activity was defined as the amount of enzyme required to inhibit NBT reduction by 50%. The specific SOD activity was expressed as U/mg protein.

### 2.4. Statistical Analysis

Unless otherwise specified, all the experiments were conducted in triplicate to obtain mean values and standard deviations. Experimental data were analyzed using SPSS software (SPSS 25 for Windows, SPSS Inc., Chicago, IL, USA). Statistical significance between means was calculated using analysis of variance (ANOVA) and Duncan’s new multiple range test at *p* < 0.05.

## 3. Results and Discussion

### 3.1. Properties of HPMC/ML Film

#### 3.1.1. Mechanical Properties

The mechanical properties of the HPMC/ML films were evaluated in terms of thickness, tensile strength (TS), and elongation at break (EAB) (Table 1). The incorporation of ML (25–100 µM) resulted in no significant differences in the mechanical performance of the composite films compared to the control HPMC film. Considering the relatively low concentration of ML in the film-forming formulations, these findings suggested that ML incorporation did not produce sufficiently pronounced changes to affect the measured mechanical response of the HPMC films under the investigated conditions. Similar findings were reported by Cabrera-Barjas et al. [28], who observed that the incorporation of low-molecular-weight compounds into HPMC films did not necessarily alter their mechanical properties. Furthermore, previous studies reported that HPMC-based edible coatings incorporating active compounds could maintain the sensory quality of coated fresh produce without adverse effects [29,30]. These findings further suggested the potential suitability of HPMC/ML as a coating material for postharvest green asparagus.

#### 3.1.2. Optical Properties

The transparency of the coating material is one of the critical factors influencing consumer acceptance of coated food products. As shown in Table 1, all the composite films exhibited transparency values ranging from 0.60 to 0.61 Abs/mm with no significant differences observed between the control HPMC film and all the other HPMC/ML films. These results suggested that the incorporation of ML did not produce detectable changes in the optical clarity of the HPMC films. The results concurred with Koka et al. [31], who reported that the incorporation of ML into HPMC matrices resulted in clear and transparent films. These findings suggested that the incorporation of ML at the investigated concentrations maintained the optical clarity of the HPMC films, indicating their potential suitability for edible coating applications where transparency is desirable.

#### 3.1.3. Barrier Properties

The barrier properties of the HPMC/ML films were evaluated by determining the OTR and WVTR (Table 1). The HPMC control film exhibited the lowest OTR (1107.08 ± 2.71 cc/m^2^/day). The incorporation of ML led to a significant increase in OTR, peaking in the HPMC/ML100 formulation at 1202.45 ± 1.65 cc/m^2^/day. The oxygen transport behavior of polymer films may be considered within the framework of free-volume theory, which describes the relationship between molecular packing, accessible free volume, and gas diffusion through polymer matrices [32]. Previous research by Laksmana et al. [33] further demonstrated the relevance of fractional free volume to diffusion behavior in HPMC films. Although ML incorporation did not significantly affect the mechanical properties of the films (Section 3.1.1), molecular transport properties may be sensitive to subtle changes in the local polymer environment that are not necessarily reflected in bulk mechanical performance. Similarly, Choi, Singh and Lee [29] reported an increase in OTR following the incorporation of essential oils (0.5–2.0%) into HPMC films, which they associated with changes in film microstructure and the formation of void spaces. These findings demonstrated that the incorporation of active compounds could influence oxygen transport through HPMC matrices. Accordingly, the increase in OTR observed in the present study may be associated with subtle changes in the local packing arrangement of HPMC chains, potentially increasing the accessibility of free-volume pathways for oxygen diffusion.

Conversely, the WVTR was highest in the control HPMC film (17.97 ± 0.24 g/m^2^/day). The addition of ML resulted in a significant reduction in water vapor transmission, reaching a minimum of 17.21 ± 0.24 g/m^2^/day. Unlike oxygen transport, water vapor transmission through hydrophilic polymer matrices is governed by both moisture sorption and molecular diffusion, which are influenced by polymer–water interactions and the physicochemical characteristics of the incorporated compounds [17,34]. Sánchez-González, Vargas, González-Martínez, Chiralt and Cháfer [34] similarly reported that incorporating tea tree essential oil into HPMC films reduced water vapor permeability and moisture sorption capacity, demonstrating the potential influence of hydrophobic compounds on moisture transport through HPMC matrices. In the present study, the relatively hydrophobic character of ML may have subtly influenced local interactions between water molecules and the HPMC matrix, potentially reducing water affinity and contributing to the observed decrease in WVTR [35]. The observed reduction in WVTR may provide particularly advantageous for coating asparagus, as it may aid in minimizing moisture loss and retarding shriveling during storage.

Nevertheless, it should also be noted that the OTR and WVTR measurements were conducted under controlled testing conditions, which differ from the storage condition of asparagus. Because temperature and RH can substantially influence the diffusion and permeability of hydrophilic polymer films, such as HPMC-based materials [17,33], the measured OTR and WVTR values in this study were interpreted as comparative indicators of the barrier performance of the free-standing films under the specified testing conditions rather than direct quantitative predictions of the barrier properties of the coatings during cold storage.

### 3.2. Effect of HPMC/ML on Asparagus Quality During Cold Storage

#### 3.2.1. Appearance and Color

**Figure 1:**
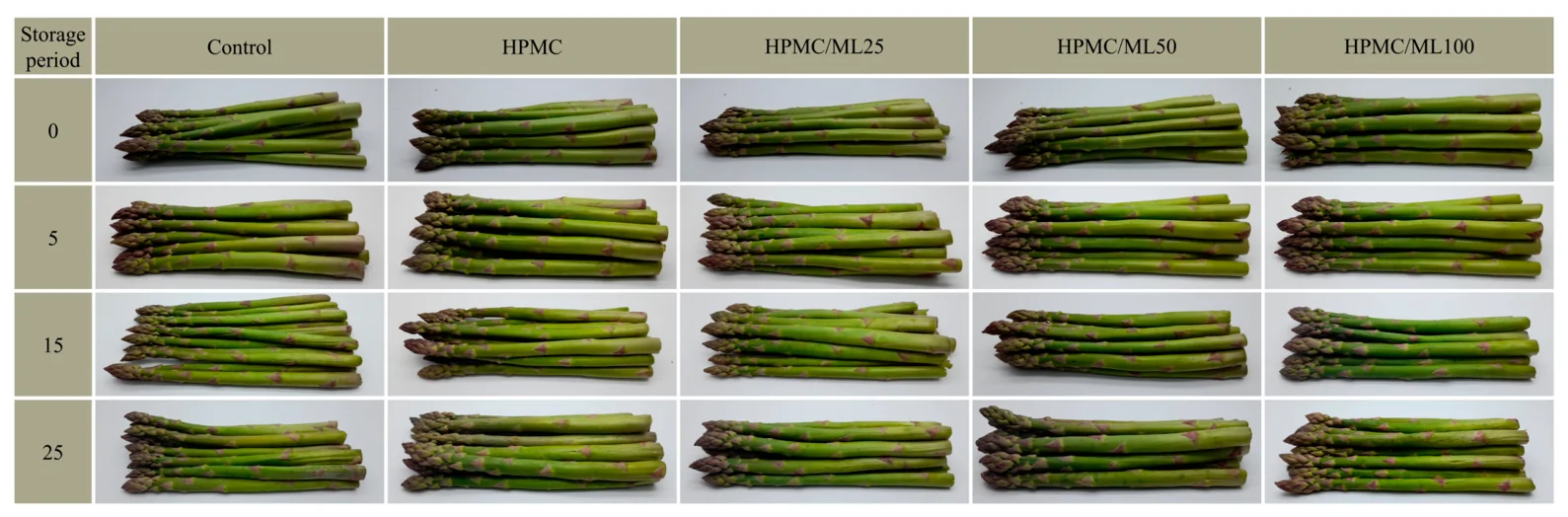
Visual appearance of green asparagus coated with HPMC/ML containing varying concentrations of melatonin (0, 25, 50, and 100 µM) during cold storage at 4 °C.

The visual appearance of uncoated (control) and HPMC/ML-coated green asparagus over 25 days of storage is shown in Figure 1. As observed, the HPMC- and HPMC/ML-coated asparagus exhibited no pronounced visual differences from the control asparagus at the beginning of storage (day 0), suggesting that the coating treatments did not substantially alter their initial appearance. Throughout the storage period, all the samples exhibited a color shift from the original fresh green toward yellow. The control asparagus showed the most pronounced yellowing, followed by the HPMC-coated samples, whereas the HPMC/ML treatments exhibited better retention of green appearance. At the end of storage, surface wrinkling was evident, particularly in the middle and basal regions, with the control group exhibiting the most severe shriveling. Coating applications appeared to mitigate this wrinkling, with HPMC/ML50 showing the least apparent wrinkling. However, HPMC/ML100 exhibited greater wrinkling, comparable to HPMC alone, indicating that increasing the ML concentration beyond 50 µM did not necessarily provide additional improvement in overall appearance preservation. This response may reflect the concentration-dependent, biphasic (hormetic) effects of ML. At an effective concentration, ML may enhance antioxidant defense and regulate reactive oxygen species (ROS) signaling, thereby maintaining cellular membrane integrity and tissue turgor. However, increasing ML beyond this concentration range may not provide additional physiological benefits, potentially due to saturation or altered regulation of these protective responses [36,37]. Nevertheless, considering that the coating was applied only to the basal part of the asparagus spears, the observed preservation of overall appearance may be partially attributed to the protection of the directly coated region. Furthermore, the preservation effects observed beyond this region may involve physiological responses associated with ML treatment, as previous research demonstrated that localized ML application could induce systemic responses in untreated plant tissues [38].

**Figure 2:**
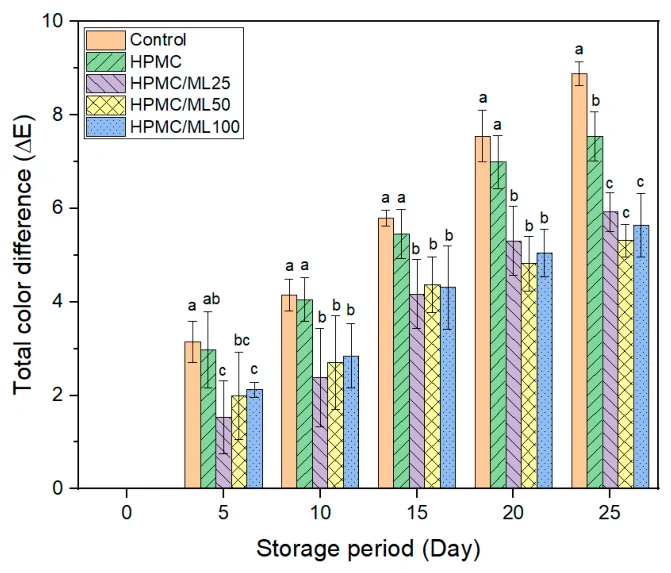
Total color difference measured at the middle region of green asparagus coated with HPMC/ML during cold storage. Different letters indicate significant differences among treatments within the same storage period (*p* < 0.05).

The objective color measurements were analyzed to quantify these color changes, with the results presented in Table 2. Consistent with visual observations, the L* value decreased while the a* and b* values increased during storage, indicating a loss of greenness and an increase in yellowness. The total color difference (ΔE, Figure 2) further confirmed that the color parameters of the control asparagus exhibited the greatest color change, with a ΔE value of 8.88 ± 0.25. The HPMC coating retarded this change, with a ΔE value of 7.54 ± 0.52. The incorporation of ML significantly delayed color changes, with ΔE values of 5.92 ± 0.41, 5.31 ± 0.35, and 5.64 ± 0.69 for HPMC/ML25, HPMC/ML50, and HPMC/ML100, respectively. Changes in the a* and b* values were directly attributed to the degradation of chlorophyll and the unmasking of carotenoids, leading to a decrease in greenness and an increase in yellowness intensity, contributing to the change in ΔE [9,39]. Notably, no significant difference in ΔE was observed between HPMC/ML50 and HPMC/ML100 at the end of storage, indicating that increasing the ML concentration beyond 50 µM did not provide additional benefits in overall color preservation. These findings suggested that HPMC/ML50 offered a favorable balance between ML concentration and appearance preservation, potentially providing a more economical formulation by reducing the amount of ML required without compromising the measured color preservation performance.

#### 3.2.2. Chlorophyll Content

**Figure 3:**
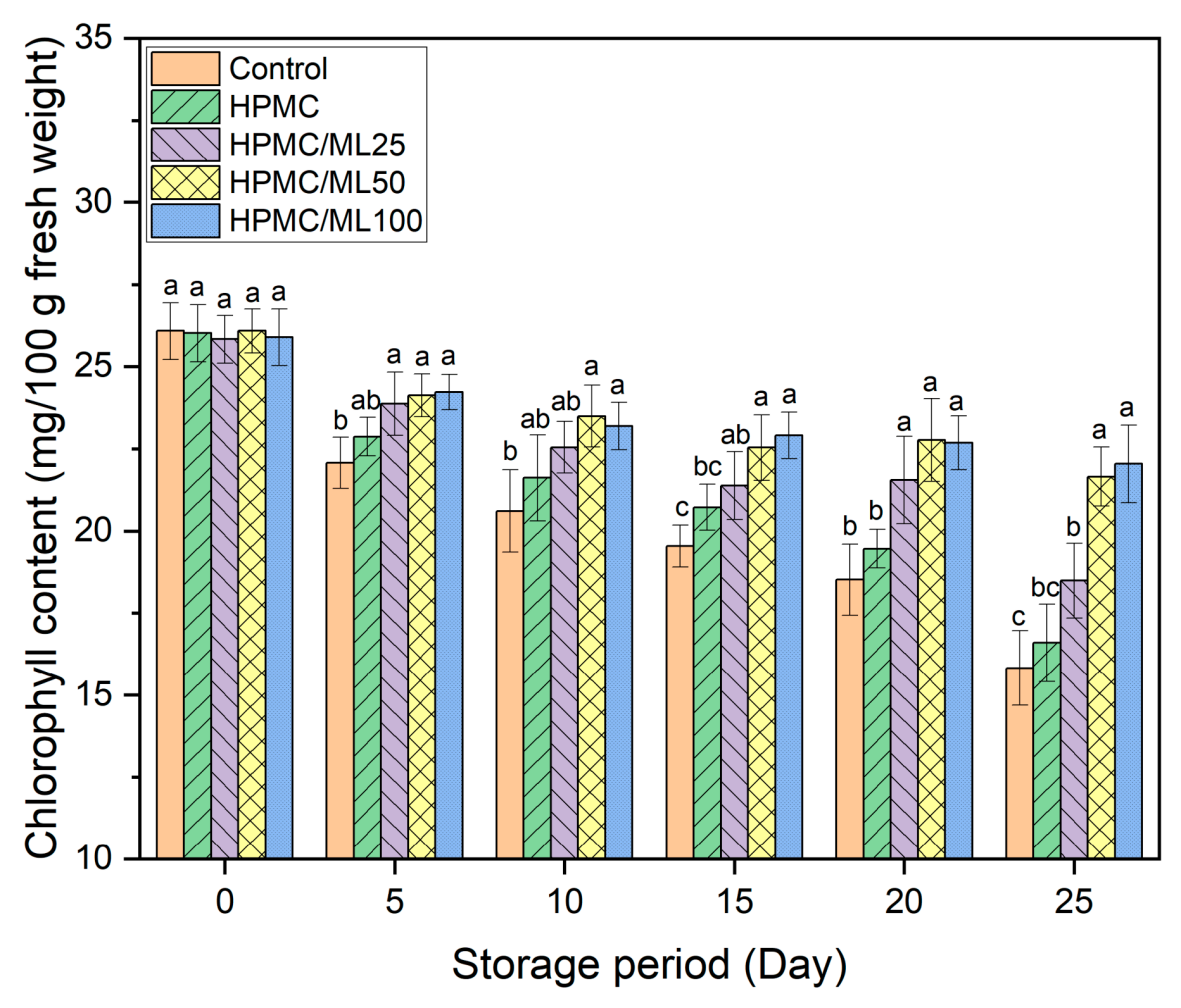
Chlorophyll content of green asparagus coated with HPMC/ML during cold storage. Different letters indicate significant differences among treatments within the same storage period (*p* < 0.05).

The chlorophyll content is one of the primary indicators of freshness in green asparagus. After harvesting, the chlorophyll content decreases gradually with storage time, with this decrease accelerated by the activity of chlorophyll-degrading enzymes, such as chlorophyllase and Mg-dechelatase, as well as oxidative stress [21,40]. The chlorophyll content of asparagus during storage is illustrated in Figure 3. The initial chlorophyll content of all the samples ranged from 25.83 to 26.08 mg/100 g fresh weight. The control asparagus presented the highest loss in chlorophyll content, decreasing by 39.94% of the initial value after 25 days of storage. The HPMC coating significantly reduced this loss to 36.21% relative to the initial value. The incorporation of ML into the HPMC matrix further preserved chlorophyll content, with losses of 28.41%, 16.98%, and 14.87% for HPMC/ML25, HPMC/ML50, and HPMC/ML100, respectively, relative to their initial values. The preservation of green pigment in the HPMC/ML-coated samples was attributed to the regulatory role of ML, which downregulated the key chlorophyll catabolism enzymes and other senescence-promoting genes and scavenged reactive oxygen species that damaged chloroplasts [12]. The decrease in chlorophyll content was consistent with the observed color changes, indicating that HPMC/ML coatings effectively delayed pigment degradation of green asparagus during cold storage.

Interestingly, although HPMC/ML100 exhibited the lowest chlorophyll loss, no significant difference in chlorophyll content was observed between HPMC/ML50 and HPMC/ML100 at the end of storage. Furthermore, the slightly higher chlorophyll retention of HPMC/ML100 did not correspond to additional improvement in overall visual appearance or total color difference (Figure 2). This apparent discrepancy may be explained by the fact that chlorophyll content specifically reflects pigment preservation, whereas overall visual appearance is influenced by multiple quality attributes, including surface color, tissue hydration, and structural changes associated with senescence [41]. Therefore, the preservation of chlorophyll alone may not fully represent the extent of overall visual deterioration. These findings suggested that increasing the ML concentration beyond 50 µM provided limited additional benefits in pigment retention without a corresponding improvement in overall appearance. Consequently, HPMC/ML50 was considered a favorable formulation, providing comparable chlorophyll and visual preservation performance.

#### 3.2.3. Weight Loss

**Figure 4:**
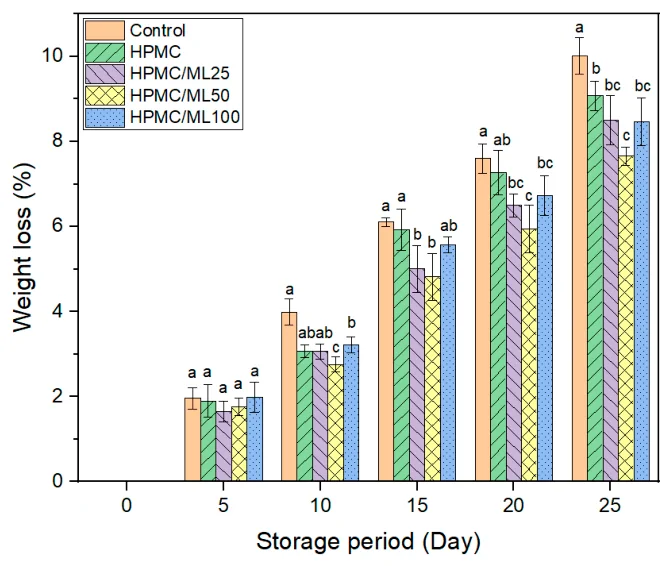
Weight loss of green asparagus coated with HPMC/ML during cold storage. Different letters indicate significant differences among treatments within the same storage period (*p* < 0.05).

Asparagus is susceptible to substantial water loss through transpiration, which contributes to significant water loss, physiological deterioration, and shriveling [42]. Minimizing weight loss is crucial for green asparagus marketability, with losses <6% generally considered acceptable [43]. The weight loss of all the asparagus samples after coating treatment gradually increased during storage (Figure 4). By the end of the 25-day storage period, the control asparagus exhibited the highest weight loss, reaching 10.00 ± 0.43%. The HPMC coating alone significantly reduced this weight loss to 9.08 ± 0.35%. The combination of HPMC and ML further improved water retention, with final weight losses of 8.50 ± 0.58%, 7.66 ± 0.21%, and 8.46 ± 0.56% for the 25 µM, 50 µM, and 100 µM ML, respectively. Notably, HPMC/ML50 exhibited a mean value of weight loss slightly below the 6% acceptability threshold on day 20, whereas the other treatments showed weight loss exceeding this level. The reduction in weight loss may be partially attributed to the moisture barrier provided by the HPMC coating, which could restrict water vapor transfer from the directly coated basal region. The incorporation of ML also resulted in a reduction in WVTR (Table 1), suggesting a potential improvement in the moisture barrier performance of the films. In addition to the physical barrier effect, ML may have contributed to postharvest preservation through its reported ability to alleviate oxidative stress and maintain cellular membrane integrity, as demonstrated in previous studies on fresh produce [13,44]. Interestingly, increasing the ML concentration from 50 to 100 µM did not further reduce weight loss, with HPMC/ML100 exhibiting a slightly higher value. This observation may be associated with the concentration-dependent physiological responses of plants to exogenous ML, for which increasing concentrations do not necessarily produce proportional improvements in stress tolerance or postharvest preservation. This observation was consistent with the observed greater apparent wrinkling in HPMC/ML100 compared to HPMC/ML50 (Figure 1), suggesting that differences in tissue water status may have contributed to the observed variation in surface wrinkling. Similar results have been reported for postharvest ML treatment of horticultural crops, suggesting that the effect of ML was concentration-dependent, with an effective optimal range [37,45]. Therefore, HPMC/ML50 was considered the preferred formulation for reducing weight loss of asparagus during cold storage.

#### 3.2.4. Texture

**Figure 5:**
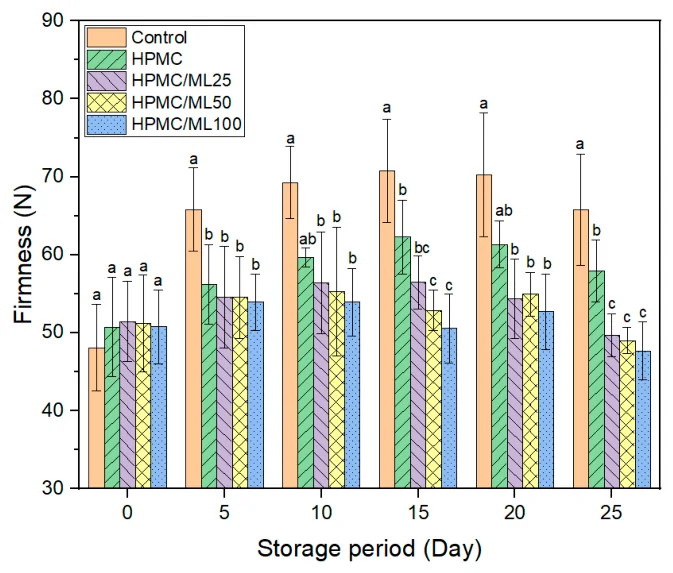
Firmness of the basal region of green asparagus coated with HPMC/ML during cold storage. Different letters indicate significant differences among treatments within the same storage period (*p* < 0.05).

Texture is one of the critical quality parameters influencing consumer acceptance of asparagus [5]. The firmness of the basal region of the asparagus spear during storage is illustrated in Figure 5. All the samples exhibited initial firmness ranging from 48.06 to 51.46 N. The firmness of the control asparagus increased significantly, peaking at 70.79 ± 6.66 N on day 15. This initial toughening is a typical postharvest response in asparagus, attributed to secondary cell wall thickening and the lignification of pericyclic fibers [46]. The application of HPMC delayed this increase, peaking at 62.28 ± 4.77 N on day 15. This delay was enhanced by the incorporation of ML, with the 50 µM and 100 µM ML samples maintaining the lowest peak firmness at 52.90 ± 2.56 and 50.60 ± 4.42 (without a significant difference), respectively. The results indicate that the application of HPMC/ML coatings, particularly at 50 and 100 µM ML, significantly attenuated the initial increase in firmness associated with postharvest toughening of green asparagus during cold storage.

Interestingly, a decline in firmness was observed during the later stages of storage (after day 15). This trend may indicate a shift in the dominant factors influencing tissue mechanical properties, from lignification-associated toughening during the early storage period to senescence-related textural deterioration during prolonged storage. A similar pattern was reported by Song et al. [47], who observed an initial increase in the firmness of the basal region of green asparagus stored at 4 °C, followed by a subsequent decline despite continuous lignin accumulation. These findings suggest that asparagus firmness is not exclusively determined by lignification but is also influenced by changes in cell-wall composition and tissue water status. Furthermore, Huyskens-Keil et al. [48] reported that reduced cutting force during asparagus storage was associated with changes in water relations and pectic substances, suggesting that alterations in cell-wall integrity may contribute to declining tissue strength. Consistent with the progressive weight loss observed during storage (Figure 4), the results suggest that changes in tissue hydration and turgor-related mechanical properties may have contributed to the late-stage decline in firmness.

#### 3.2.5. Lignin Content

**Figure 6:**
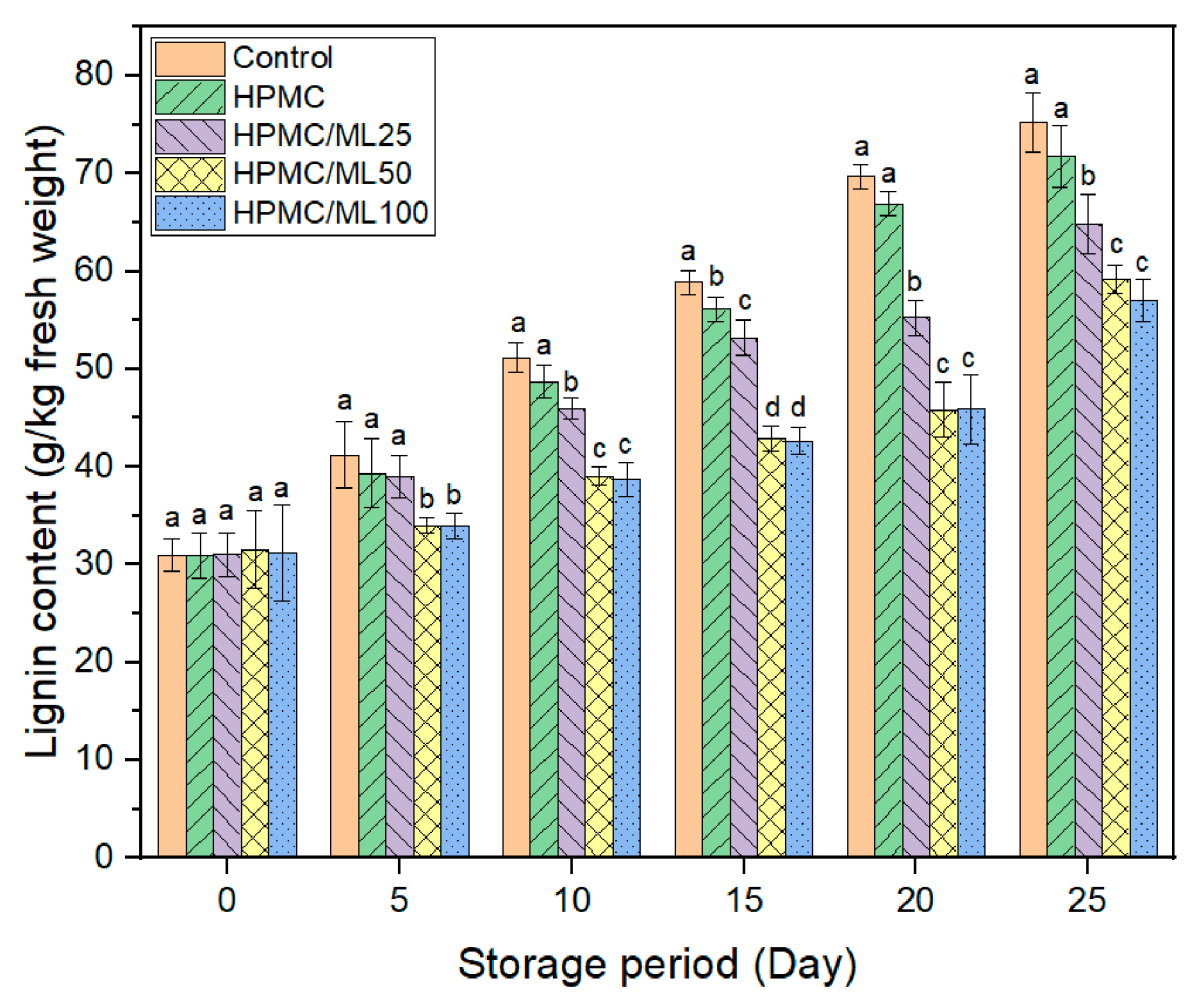
Lignin content of green asparagus coated with HPMC/ML during cold storage. Different letters indicate significant differences among treatments within the same storage period (*p* < 0.05).

Lignin is a complex phenylpropanoid polymer that imparts structural rigidity to plant tissues [4,5]. Consistent with the initial increase in firmness, lignin content accumulated in all samples throughout the storage period (Figure 6). At the very end of the storage period (day 25), the control asparagus exhibited the highest accumulation of lignin, reaching 75.25 ± 3.03 g/kg fresh weight, reflecting extensive postharvest lignification and secondary cell wall thickening over time. This accumulation of lignin explained the increase in firmness observed in Section 3.2.4. On the contrary, asparagus coated with HPMC exhibited significantly lower lignin levels at the end of storage, at 71.73 ± 3.16 g/kg fresh weight. Notably, the HPMC/ML-coated asparagus showed reduced lignin accumulation, with the lowest lignin contents observed in HPMC/ML50 and HPMC/ML100 at 59.22 ± 1.40 and 57.05 ± 2.12 g/kg fresh weight, respectively, at the end of storage. Although HPMC/ML100 exhibited a slightly lower lignin content, no significant difference was observed between these two formulations, indicating that increasing the ML concentration beyond 50 µM did not provide an additional significant improvement in inhibiting lignin accumulation. Consistent with the firmness results (Figure 5), these findings further supported the effectiveness of both formulations in delaying postharvest toughening, with HPMC/ML50 being considered the preferred formulation for reducing postharvest lignification under the investigated conditions.

Nevertheless, although lignin content continued to increase throughout storage, firmness declined after day 15, suggesting that lignification alone could not fully explain the changes in tissue mechanical properties. This observation is consistent with previous findings in postharvest asparagus, where reductions in firmness occurred despite continued lignin accumulation [47]. The late-stage decline in firmness may therefore reflect the combined influence of senescence-related changes in tissue water status, cellular turgor, and cell-wall integrity, which could affect mechanical resistance independently of lignin accumulation [41].

#### 3.2.6. Lignification-Related Enzymes

**Figure 7:**
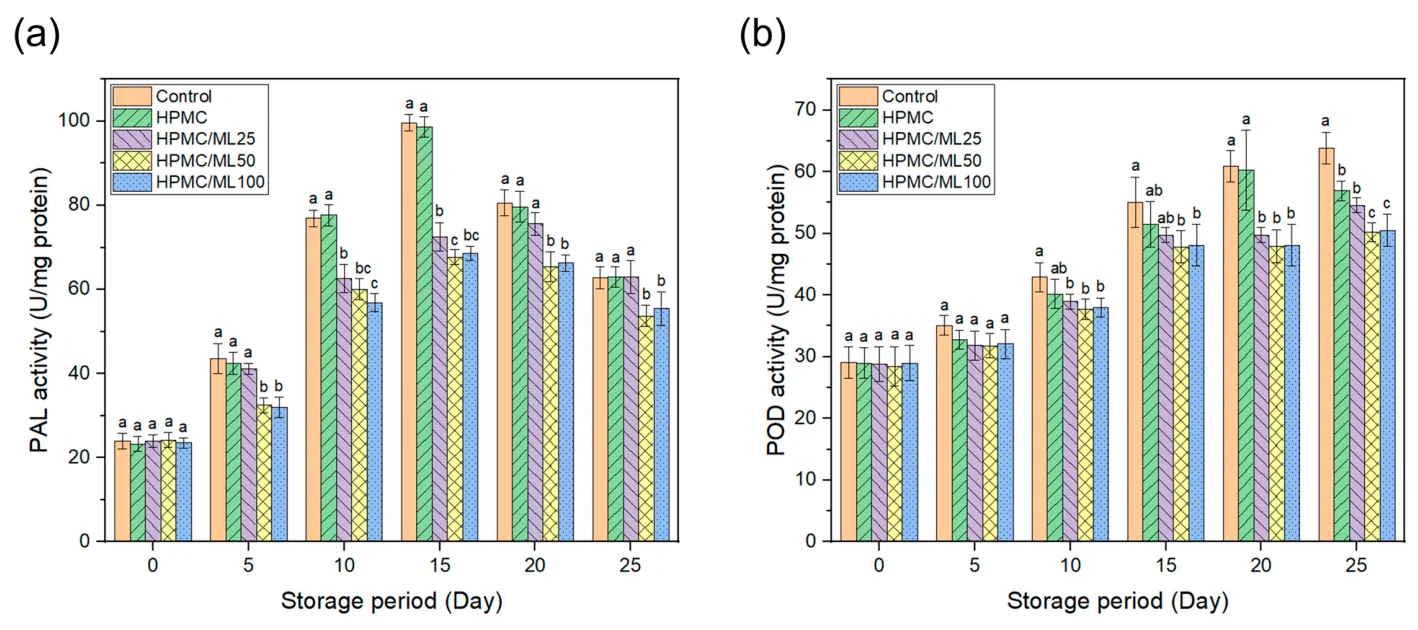
Lignification-related enzyme activity of green asparagus coated with HPMC/ML during cold storage: (**a**) phenylalanine ammonia-lyase (PAL) activity, and (**b**) peroxidase (POD) activity. Different letters indicate significant differences among treatments within the same storage period (*p* < 0.05).

The activities of PAL and POD were analyzed to understand the metabolic regulation of the observed lignin accumulation. PAL is the key enzyme providing precursors for the phenylpropanoid pathway, while POD catalyzes the polymerization of lignin monomers [26,40,49]. As shown in Figure 7a, PAL activity increased until day 15 and then declined, suggesting that the phenylpropanoid precursor supply was most active during the early storage period before progressively decreasing. POD activity increased continuously until the end of the storage period (Figure 7b), indicating a sustained potential for lignin polymerization. This continuous POD activity was consistent with the progressive lignin accumulation observed in Figure 6. Asparagus coated with HPMC/ML exhibited consistently lower PAL and POD activities than the control during storage, with the 50 µM and 100 µM ML samples showing the lowest values throughout storage. Notably, no significant differences in PAL and POD activities were observed between HPMC/ML50 and HPMC/ML100 at the end of storage, consistent with their comparable inhibition of lignin accumulation (Figure 6). These findings indicated that increasing the ML concentration beyond 50 µM did not provide additional significant benefits in suppressing lignification-related enzyme activities. Therefore, HPMC/ML50 may represent an appropriate formulation, with a lower ML concentration while providing comparable inhibition of lignification. The reduction in PAL and POD activities suggested that HPMC/ML coatings attenuated phenylpropanoid flux and limited oxidative polymerization capacity, thereby restraining lignin accumulation and contributing to the lower tissue firmness observed in HPMC/ML-coated asparagus. This modulation of the PAL- and POD-associated lignification processes concurred with the transcriptomic evidence that melatonin redirected phenylpropanoid metabolism and suppressed lignin-related oxidative reactions during postharvest storage [50]. These findings were also consistent with previous studies reporting that exogenous melatonin inhibited lignification enzymes in postharvest asparagus [15].

#### 3.2.7. Hydrogen Peroxide (H_2_O_2_) Content

**Figure 8:**
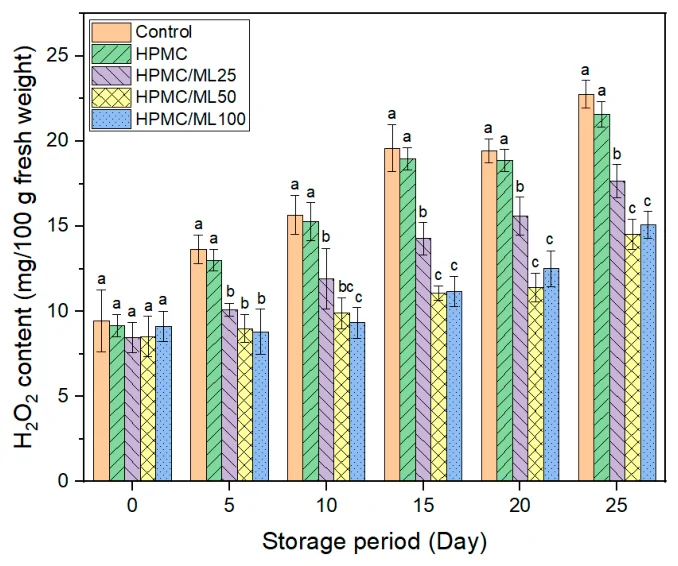
Hydrogen peroxide (H_2_O_2_) content of green asparagus coated with HPMC/ML during cold storage. Different letters indicate significant differences among treatments within the same storage period (*p* < 0.05).

To better understand asparagus senescence, the oxidative status of the tissue was evaluated via the H_2_O_2_ content. H_2_O_2_ is a reactive oxygen species (ROS) that induces lipid peroxidation and membrane damage, serving as a key marker of senescence [51]. As illustrated in Figure 8, the H_2_O_2_ content increased continuously throughout storage. The control asparagus presented the highest H_2_O_2_ content throughout storage, reaching 22.76 ± 0.83 mg/100 g fresh weight. The application of HPMC/ML significantly mitigated H_2_O_2_ accumulation, with HPMC/ML50 showing the lowest level at 14.53 ± 0.91 mg/100 g fresh weight at the end of storage. Although HPMC/ML100 effectively suppressed H_2_O_2_ accumulation, no significant difference was observed between HPMC/ML50 and HPMC/ML100, indicating that increasing the ML concentration beyond 50 µM did not further enhance the suppression of H_2_O_2_ accumulation. These findings further supported HPMC/ML50 as a favorable formulation for the mitigation of H_2_O_2_ accumulation of green asparagus during cold storage. The H_2_O_2_ results corresponded to the chlorophyll content (Section 3.2.2), because H_2_O_2_ is involved in chlorophyll bleaching when catalyzed by POD. Thus, the lower H_2_O_2_ content led to a reduction in chlorophyll degradation, which delayed yellowing [5]. Previous research reported that exogenous ML, as an efficient ROS scavenger, inhibited ROS accumulation, thereby delaying lignification and chlorophyll degradation in Pak choi and bamboo shoots [6,11]. The excessive accumulation of ROS may contribute to membrane lipid peroxidation and the deterioration of cellular integrity during postharvest senescence [41]. Such changes, together with alterations in tissue water status and cell-wall integrity, could influence the mechanical resistance of asparagus and potentially contribute to the late-stage decline in firmness despite continued lignin accumulation, as discussed in Section 3.2.4 and Section 3.2.5.

#### 3.2.8. Antioxidant-Related Enzymes

**Figure 9:**
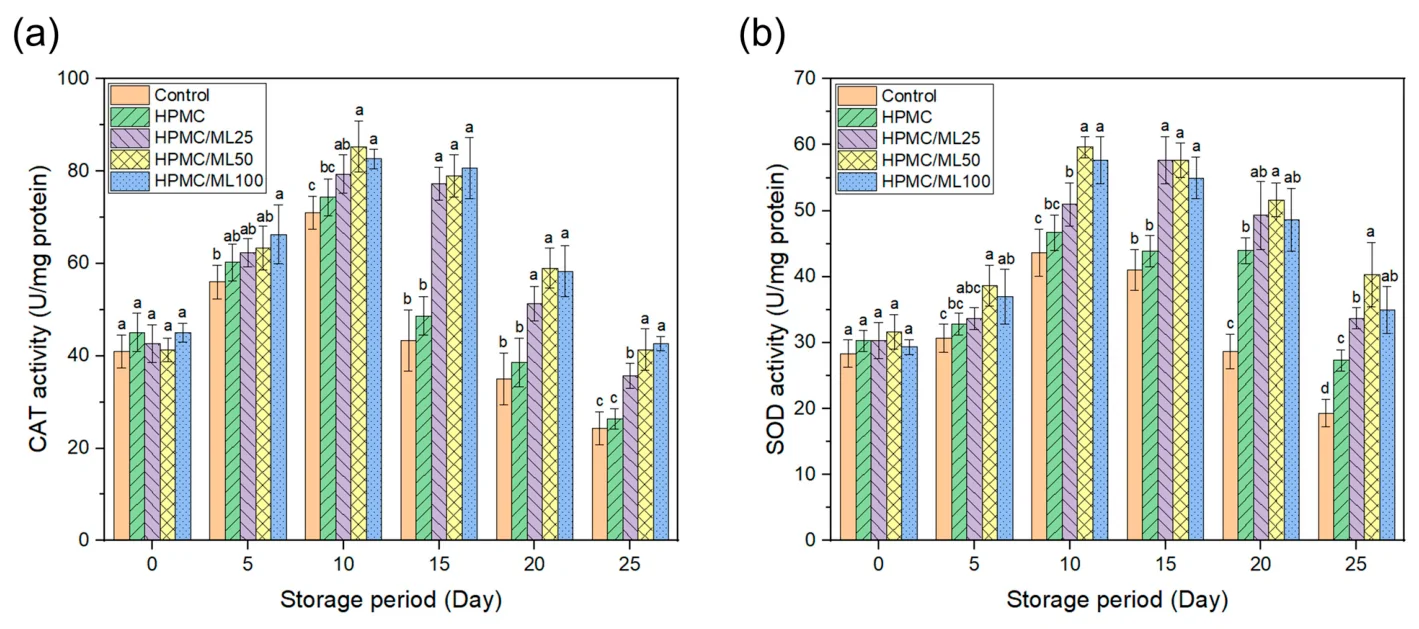
Antioxidant-related enzyme activity of green asparagus coated with HPMC/ML during cold storage: (**a**) catalase (CAT) activity, and (**b**) superoxide dismutase (SOD) activity. Different letters indicate significant differences among treatments within the same storage period (*p* < 0.05).

Antioxidant enzymes, such as CAT and SOD, are critical to protect fruits and vegetables against the accumulation of ROS, maintaining cellular structures from oxidative damage [52]. These enzymes normally decline during senescence, and can be used to indicate the rate of green asparagus senescence during storage [41]. The CAT and SOD activities of all the samples (Figure 9a,b) exhibited similar trends, increasing during the first 10 days of storage, followed by a gradual decline. The application of HPMC/ML enhanced antioxidant enzyme activities at the initial stages of storage and maintained these activities at significantly higher levels during the late storage period. At the end of storage, HPMC/ML50 and HPMC/ML100 maintained comparable CAT activities without significant differences, whereas HPMC/ML50 exhibited the highest SOD activity. These results indicated that increasing the ML concentration beyond 50 µM did not provide additional improvements in antioxidant enzyme activities. Together with the comparable suppression of H_2_O_2_ accumulation observed for both treatments (Figure 8), these findings further supported the suitability of HPMC/ML50 for maintaining the antioxidant defense system of green asparagus. The sustained enzymatic activity was attributed to the multifunctional role of ML as a broad-spectrum antioxidant, acting through the direct scavenging of ROS and the modulation of antioxidant-related gene expression [6,45,53]. Previous research also reported that exogenous ML delayed postharvest senescence of asparagus and plants by enhancing the activities of antioxidant enzymes [14,15]. The decline in these scavenging enzymes coincided with a drop in firmness, suggesting that the loss in firmness occurred from the senescence of green asparagus. The HPMC/ML-coated asparagus retained higher CAT and SOD activity, effectively delaying oxidative damage, which protected membrane integrity and delayed senescence-related tissue collapse, thereby maintaining better textural quality at the end of storage.

## 4. Conclusions

HPMC-based edible films incorporating ML were successfully developed using the solvent casting technique, and the corresponding film-forming solutions were applied as edible coatings to the basal region of green asparagus using the dipping technique. The application of HPMC/ML coatings delayed postharvest quality deterioration of green asparagus during 25 days of cold storage, including weight loss, chlorophyll degradation, lignin accumulation, and H_2_O_2_ accumulation. These preservation effects were accompanied by reduced activities of lignification-related enzymes (PAL and POD) and enhanced antioxidant enzyme activities (CAT and SOD), contributing to the attenuation of postharvest senescence and changes in firmness. Although ML incorporation generally improved preservation performance, increasing the ML concentration beyond 50 µM did not provide additional statistically significant improvements in several key quality attributes. Therefore, considering the overall quality attributes and ML concentration, HPMC/ML50 was identified as the recommended formulation for maintaining green asparagus quality during cold storage. Nevertheless, further investigations under practical storage conditions, including sensory and microbial quality assessments, are necessary to establish its effects on commercial shelf life.

## Figures and Tables

**Table 1:** Characteristics of hydroxypropyl methylcellulose (HPMC) films containing 25 µM melatonin (HPMC/ML25), 50 µM melatonin (HPMC/ML50), and 100 µM melatonin (HPMC/ML100) *.

Sample	Thickness (µm)	TS (MPa)	EAB (%)	Transparency (Abs/mm)	OTR (cc/m^2^/Day)	WVTR (g/m^2^/Day)
HPMC	33.11 ± 1.61 ^a^	2.34 ± 0.62 ^a^	13.07 ± 2.20 ^a^	0.60 ± 0.01 ^a^	1107.08 ± 2.71 ^c^	17.97 ± 0.24 ^a^
HPMC/ML25	32.95 ± 1.73 ^a^	2.32 ± 0.58 ^a^	13.16 ± 2.05 ^a^	0.60 ± 0.01 ^a^	1114.00 ± 7.31 ^c^	17.81 ± 0.15 ^ab^
HPMC/ML50	32.62 ± 1.04 ^a^	2.34 ± 0.54 ^a^	13.15 ± 2.60 ^a^	0.60 ± 0.02 ^a^	1156.44 ± 7.03 ^b^	17.46 ± 0.29 ^bc^
HPMC/ML100	32.78 ± 1.04 ^a^	2.37 ± 0.60 ^a^	13.28 ± 2.34 ^a^	0.61 ± 0.02 ^a^	1202.45 ± 1.65 ^a^	17.21 ± 0.24 ^c^

* Data represent the mean ± SD. Different superscript letters indicate significant differences in the data within the same column.

**Table 2:** Effect of HPMC/ML coating on color attributes (L*, a*, b*) of the middle region of green asparagus during 25 days of storage at 4 °C and 85% relative humidity *.

	Storage Period (Day)					
	0	5	10	15	20	25
L*						
Control	27.76 ± 0.61 ^a,A^	25.82 ± 0.50 ^b,B^	24.34 ± 0.66 ^c,C^	23.70 ± 0.48 ^c,C^	22.68 ± 0.30 ^c,D^	22.80 ± 0.33 ^c,D^
HPMC	27.60 ± 0.43 ^a,A^	26.84 ± 0.52 ^a,A^	24.56 ± 0.84 ^c,B^	23.88 ± 0.65 ^bcBC^	23.12 ± 0.41 ^bc,CD^	22.98 ± 0.54 ^bc,D^
HPMC/ML25	27.60 ± 0.37 ^a,A^	26.82 ± 0.64 ^a,B^	26.70 ± 0.67 ^a,B^	24.50 ± 0.64 ^ab,C^	24.06 ± 0.44 ^a,CD^	23.44 ± 0.36 ^ab,D^
HPMC/ML50	27.18 ± 0.51 ^a,A^	26.54±0.84 ^ab,AB^	26.26±0.61 ^ab,B^	24.70 ± 0.35 ^a,C^	23.76 ± 0.73 ^ab,D^	23.70 ± 0.29 ^a,D^
HPMC/ML100	27.10 ± 0.29 ^a,A^	26.46 ± 0.72 ^ab,AB^	25.74 ± 0.61 ^b,B^	24.34 ± 0.38 ^abc,C^	23.52 ± 0.83 ^ab,D^	23.18 ± 0.51 ^abc,D^
a*						
Control	−2.50 ± 0.20 ^a,D^	−2.54 ± 0.11 ^ab,D^	−1.74 ± 0.21 ^ab,C^	0.00 ± 0.32 ^a,B^	0.12 ± 0.51 ^a,B^	2.78 ± 0.31 ^a,A^
HPMC	−2.50 ± 0.46 ^a,D^	−2.98 ± 0.13 ^c,D^	−1.86 ± 0.11 ^b,C^	−0.64 ± 0.88 ^b,B^	−0.44 ± 0.29 ^b,B^	1.08 ± 0.11 ^b,A^
HPMC/ML25	−2.50 ± 0.46 ^a,C^	−2.50 ± 0.11 ^a,C^	−1.60 ± 0.16 ^a,B^	−1.46 ± 0.24 ^c,B^	−0.43 ± 0.08 ^b,A^	−0.28 ± 0.40 ^c,A^
HPMC/ML50	−2.58 ± 0.55 ^a,B^	−2.85 ± 0.41 ^bc,B^	−2.48 ± 0.15 ^c,B^	−0.86 ± 0.13 ^b,A^	−0.68 ± 0.33 ^b,A^	−0.78 ± 0.33 ^d,A^
HPMC/ML100	−2.62 ± 0.61 ^a,C^	−2.54 ± 0.21 ^ab,C^	−1.70 ± 0.16 ^ab,B^	−1.76 ± 0.11 ^c,B^	−0.80 ± 0.09 ^b,A^	−0.60 ± 0.16 ^cd,A^
b*						
Control	29.26 ± 0.42 ^a,D^	31.68 ± 0.55 ^a,C^	31.38 ± 0.49 ^a,C^	32.48 ± 0.45 ^a,B^	34.14 ± 0.80 ^a,A^	34.34 ± 0.71 ^a,A^
HPMC	28.74 ± 0.59 ^a,C^	31.52 ± 0.89 ^ab,B^	31.02 ± 1.20 ^a,B^	32.08 ± 0.79 ^a,B^	33.64 ± 0.93 ^ab,A^	33.46 ± 0.78 ^ab,A^
HPMC/ML25	29.30 ± 0.80 ^a,D^	30.46 ± 0.83 ^b,CD^	31.20 ± 1.08 ^a,BC^	31.78 ± 0.84 ^a,AB^	32.54 ± 1.15 ^bc,A^	32.82 ± 0.67 ^bc,A^
HPMC/ML50	28.94 ± 0.99 ^a,C^	30.60 ± 0.96 ^b,B^	31.32 ± 1.26 ^a,AB^	32.04 ± 0.81 ^a,A^	31.70 ± 0.37 ^c,AB^	32.48 ± 0.58 ^c,A^
HPMC/ML100	29.12 ± 0.72 ^a,D^	31.03 ± 0.24 ^ab,C^	31.26 ± 1.06 ^a,BC^	32.24 ± 1.14 ^a,AB^	32.04 ± 0.72 ^c,ABC^	32.62 ± 0.59 ^bc,A^

* Data represent the mean ± SD. Different lowercase superscript letters indicate significant differences among treatments within the same storage day (same column), whereas different uppercase superscript letters indicate significant differences among storage days within the same treatment group (same row) (*p* < 0.05).

## Data Availability

The original contributions presented in this study are included in the article. Further inquiries can be directed to the corresponding author.

## Abbreviations

The following abbreviations are used in this manuscript:

CAT	Catalase
H_2_O_2_	Hydrogen peroxide
HPMC	Hydroxypropyl methylcellulose
HPMC/ML	HPMC-based edible coating incorporating ML
ML	Melatonin
OTR	Oxygen transmission rate
PAL	Phenylalanine ammonia-lyase
POD	Peroxidase
ROS	Reactive oxygen species
SOD	Superoxide dismutase
WVTR	Water vapor transmission rate
